# Asymmetric Dimethylarginine Vibrational Spectroscopy Spectra and Density Functional Theory Model

**DOI:** 10.3390/s25226818

**Published:** 2025-11-07

**Authors:** Luis Pablo Canul-Solís, Ma. del Carmen Rodríguez-Aranda, Emmanuel Rivera-Pérez, Alejandra Ortiz-Dosal, Edgar Guevara, Erick Osvaldo Martínez-Ruiz, Luis Carlos Ortiz-Dosal, Adán Reyes-Reyes, Eleazar Samuel Kolosovas-Machuca

**Affiliations:** 1 Facultad de Ciencias, Universidad Autónoma de San Luis Potosí, Av. Parque Chapultepec 1570, San Luis Potosí 78295, San Luis Potosí, Mexico; luis.canulvii@gmail.com (L.P.C.-S.); carmen.aranda@uaslp.mx (M.d.C.R.-A.); emmanuel.perez@uaslp.mx (E.R.-P.); alejandra.ortiz@uaslp.mx (A.O.-D.); edgar.guevara@uaslp.mx (E.G.);; 2 Coordinación para la Innovación y Aplicación de la Ciencia y la Tecnología (CIACYT), Universidad Autónoma de San Luis Potosí, Av. Sierra Leona 550, San Luis Potosí 78210, San Luis Potosí, Mexico; 3 Secretaría de Ciencia, Humanidades, Tecnología e Innovación, Universidad Autónoma de San Luis Potosí, Av. Parque Chapultepec 1570, San Luis Potosí 78295, San Luis Potosí, Mexico; 4 Centro de Investigación en Química Aplicada (CIQA), Blvd. Enrique Reyna 140, Saltillo 25294, Coahuila, Mexico; erick.martinez@ciqa.edu.mx; 5 Maestría en Ciencia e Ingeniería de los Materiales (MCIM-UAZ), Universidad Autónoma de Zacatecas, 801 López Velarde St. 9800, Zacatecas 98160, Zacatecas, Mexico; ortiz.dosal.lc@uaz.edu.mx

**Keywords:** N^G^, N^G^-dimethylarginine (ADMA), Raman spectroscopy, FT-IR spectroscopy, Density Functional Theory (DFT), biomarkers

## Abstract

**Highlights:**

**What are the main findings?**

- First comprehensive vibrational characterization of isolated NG, NG-dimethylarginine (ADMA) using Raman and FT-IR spectroscopy.
- Experimental spectra were successfully correlated with DFT-based simulated spectra, achieving up to 86.67% vibrational band assignment for FT-IR and 54% for Raman spectra.
- Key vibrational modes such as N–H scissoring (1621 cm^−1^) and C=NH stretching (1667 cm^−1^) were confirmed through experimental and theoretical methods.

**What are the implications of the main findings?**

- The isolated ADMA molecule provided a clear spectral fingerprint, free from matrix effects, establishing a reference for future biomedical and diagnostic studies.
- This integrated experimental–computational approach supports the development of spectroscopic biomarkers for clinical diagnostics related to cardiovascular diseases.

**Abstract:**

N^G^, N^G^-dimethylarginine (ADMA) is an endogenous compound that acts as a competitive inhibitor of nitric oxide synthase (NOS), thereby reducing nitric oxide (NO) production and contributing to endothelial dysfunction. This dysfunction plays a pivotal role in the development of various pathological conditions, including cardiovascular disease, chronic renal failure, and diabetes. The diminished bioavailability of NO is a critical factor in the progression of these disorders, and alterations in ADMA levels have emerged as significant predictors of cardiovascular events and mortality. In this study, we investigated the molecular characteristics of ADMA using a combined approach of Raman and Fourier Transform Infrared (FT-IR) spectroscopy, complemented by computational simulations with the GaussView 5.0.8 and Gaussian 09 software suite. Experimental Raman and FT-IR spectra were acquired and compared with simulated spectra generated through Density Functional Theory (DFT) calculations. This comparative analysis enabled precise vibrational band assignments and the identification of key molecular vibrational modes, providing valuable insights into ADMA’s structural and vibrational properties. These findings establish a comprehensive spectroscopic reference for ADMA, supporting its potential application as a biomarker in clinical diagnostics.

## 1. Introduction

Asymmetric dimethylarginine (ADMA), also referred to as N^G^, N^G^-dimethyl arginine, is an endogenous compound generated through post-translational methylation of arginine residues by protein arginine methyltransferases (PRMTs), notably PRMTs 1, 2, 3, 4, 6, and 8 [1]. ADMA is primarily eliminated via renal excretion, either unchanged or metabolized to dimethylamine and citrulline by the enzyme dimethylarginine dimethylaminohydrolase (DDAH) [2].

Functionally, ADMA acts as a competitive inhibitor of nitric oxide synthase (NOS), thereby reducing nitric oxide (NO) synthesis and impairing endothelial function. Since endothelial dysfunction is a precursor to atherosclerosis, abnormal ADMA levels have been implicated in a variety of pathologies, including peripheral arterial occlusive disease, coronary artery disease, chronic kidney disease, rheumatoid arthritis, Duchenne and Becker muscular dystrophies, attention-deficit hyperactivity disorder (ADHD), and type 1 diabetes [3]. NO plays a pivotal role in vascular homeostasis by inhibiting platelet aggregation, leukocyte adhesion, smooth muscle proliferation, and low-density lipoprotein oxidation, while also exerting anti-inflammatory effects through modulation of adhesion molecules and chemokines [4].

The clinical relevance of ADMA was first recognized in chronic renal failure [5], and since then, it has emerged as a potential biomarker for several diseases. ADMA is typically detected in biological fluids such as plasma or serum, where its concentration can be measured using chromatographic techniques or immunoassays. High-performance liquid chromatography (HPLC), often coupled with mass spectrometry (MS), remains the gold standard due to its ability to precisely separate ADMA from structurally related analogs such as symmetric dimethylarginine (SDMA) and L-arginine [6,7]. However, this technique has high operational costs and operational complexity and requires rigorous sample preparation. Although gas chromatography (GC) has also been employed, it requires derivatization for analyte volatility [8]. In contrast, enzyme-linked immunosorbent assays (ELISA) have limited specificity due to potential cross-reactivity with SDMA [9]. Other disadvantages of these analyses are the limited sensitivity and time-consuming procedures.

Vibrational spectroscopy techniques such as Raman and Fourier Transform Infrared (FT-IR) spectroscopy offer non-destructive and label-free molecular detection and characterization approaches. Raman spectroscopy is based on inelastic scattering of monochromatic light, where energy shifts corresponding to molecular vibrations provide a spectral fingerprint of the analyte. This phenomenon, first described by Raman and Krishnan in 1928, distinguishes between elastic (Rayleigh) and inelastic (Stokes and anti-Stokes) scattering processes [10,11,12]. Typically, Stokes scattering is favored in analytical applications due to the higher population of molecules in the ground vibrational state.

FT-IR spectroscopy, conversely, detects molecular vibrations via absorption of infrared radiation, contingent upon a change in the molecule’s dipole moment. This technique provides complementary information to Raman spectroscopy, which is particularly sensitive to polar functional groups. The vibrational frequencies, influenced by bond strength and atomic masses, can be modeled using the harmonic oscillator approximation [13].

Vibrational spectroscopy-based sensors offer the possibility of analyzing multiple components in a sample simultaneously with a single instrumental measurement, thereby eliminating the need for physical separation. This approach provides advantages over chromatography-based methods, including reduced reagent use, simplified sample handling, and faster analysis. However, to fully exploit its potential, the vibrational modes of compounds of biological interest must be identified individually before measuring complex biological samples.

Given the growing interest in label-free molecular detection, integrating vibrational spectroscopy techniques for ADMA quantification presents a promising avenue. This study explores the potential of Raman and FT-IR spectroscopies for identifying and characterizing ADMA, aiming to establish spectroscopic fingerprints for future diagnostic applications.

## 2. Experimental

To carry out the experimental procedures, N^G^, N^G^-dimethyl arginine dihydrochloride powder (Sigma-Aldrich, Santa Louise, MO, USA, No. CAS 220805-22-1) was procured as the target compound for analysis. Raman spectra were collected using a Horiba Jobin Yvon XploRA PLUS Raman spectrometer (Palaiseau, France) coupled to an Olympus BX41 optical microscope (Tokyo, Japan). Both 532 nm and 785 nm laser sources were utilized in a backscattering configuration for excitation, at 30 mW to avoid sample degradation. The objective lens was an ×20 long working distance (LWD) type: the power density and the area probed were equal to 14.5 mW/μm^2^ and 2 μm^2^, respectively. Finally, the diffraction grating had a groove density of 1800 lines per millimeter (gr/mm), and the spectral resolutions were equal to 0.8 cm^−1^/pixel for both laser sources. The absorbance spectrum of the compound was acquired using a Fourier Transform Infrared (FT-IR) spectrometer (Nicolet™ 6700, Thermo Fisher Scientific, Waltham, MA, USA), equipped with a Smart iTR™ Diamond Attenuated Total Reflectance (ATR) accessory (Thermo Fisher Scientific, Waltham, MA, USA). FT-IR spectra were recorded on lyophilized ADMA powder pressed directly onto the diamond crystal with uniform contact to ensure reproducibility.

The ADMA molecule is a structural analog of L-arginine with a molecular weight of 202.258 g·mol^−1^, composed of the same amino acid backbone with two methyl groups asymmetrically attached to the terminal nitrogen atom of its guanidine functional group. Single covalent bonds predominantly form the molecular structure; however, it includes two notable double bonds. These are involved in the carboxylic acid and guanidine functional groups [14].

**Figure 1:**
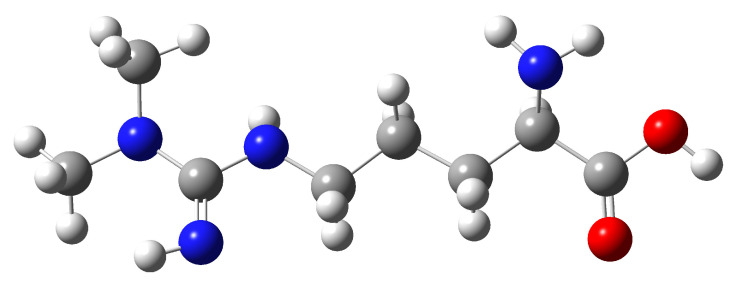
ADMA molecular structure used to optimize and calculate the simulated Raman and FT-IR spectra. Carbon atoms are shown in gray, hydrogen in white, nitrogen in blue, and oxygen in red.

A computational model of the ADMA (Figure 1) molecule was constructed using GaussView [15] 5.0.8 and Gaussian 09 [16] software. Geometry optimization and vibrational frequency calculations were performed using Density Functional Theory (DFT) with Becke’s three-parameter hybrid functional combined with the Lee–Yang–Parr correlation functional (B3LYP), employing the cc-pVDZ basis set and using a scaling factor equal to 0.96. The B3LYP approach was selected for its improved accuracy in predicting molecular vibrations and reliability [17], and the cc-pVDZ basis set was chosen for its good convergence of vibrational frequencies [18]. Simulated Raman and FT-IR spectra were derived from this model. Baseline correction was applied before data fitting to reduce fluorescence background. It was determined by fitting a polynomial curve to the measured spectrum from the points where no Raman signal was expected, and it was subtracted from the original data [19].

### 2.1. Spectral Normalization and Visualization

To facilitate direct comparison between spectra, the mathematically adjusted and simulated spectra were interpolated to match the wavenumber positions of the raw experimental spectrum using MATLAB’s (version 2019b) ‘interp1’ function. Edge effects were addressed by assigning the last interpolated value equal to the penultimate one, ensuring continuity at the boundaries. Subsequently, spectra were normalized using MATLAB’s ‘mat2gray’ function to scale intensity values to a range of [0, 1]. This normalization step eliminates variations due to differing acquisition conditions and emphasizes relative changes in spectral features [20]. For visual clarity, the spectra were plotted with vertical offsets.

### 2.2. Feature Metrics

Three distinct metrics were applied to assess spectral similarity quantitatively. Cosine Similarity, which is defined in Equation (1), calculates the cosine of the angle between two vectors in multidimensional space, reflecting orientation similarity independent of magnitude [21].

(1) $$Cosine\;similarity=\frac{A\cdot B}{\left\|A\right\|\left\|B\right\|}$$

where *A* and *B* are the spectral intensity vectors.

Pearson’s correlation coefficient evaluates the linear relationship between two spectral intensity profiles. It quantifies how one spectrum covaries with another [22]. It was determined using Equation (2).

(2) $$r=\frac{\sum_{i=1}^{n}\left(A_{i}-\bar{A}\right)\left(B_{i}-\bar{B}\right)}{\sqrt{{\sum_{i=1}^{n}\left(A_{i}-\bar{A}\right)}^{2}}\sqrt{{\sum_{i=1}^{n}\left(B_{i}-\bar{B}\right)}^{2}}}$$

where $A_{i}$ and $B_{i}$ are intensity values, and $\bar{A}$ and $\bar{B}$ are the mean intensities of spectra *A* and *B*, respectively.

Where ∆A and ∆B represent the first differences in the spectral intensity vectors, pairwise similarity values were computed for all spectra. Pearson’s correlation coefficient ranges from −1 to 1; cosine similarity ranges from −1 to 1 (typically 0 to 1 for non-negative spectra). In both, 1 denotes maximum similarity in spectral shape [23].

## 3. Results and Discussion

A numerical model of the molecule was constructed using Density Functional Theory (DFT) with Becke’s three-parameter hybrid functional combined with the Lee–Yang–Parr correlation functional (B3LYP), employing the cc-pVDZ basis set.

Although the Raman spectrum of ADMA has been previously reported by Malyshev et al. [24], no detailed vibrational mode assignments were included, and the infrared spectrum of ADMA had not been documented prior to this study. Specifically, Malyshev et al. reported a 785 nm Raman spectrum of powdered ADMA over a restricted window (~700–1200 cm^−1^), without a complete set of mode assignments. In contrast, here we provide Raman (532/785 nm) for isolated ADMA spanning a broader range (200–1750 cm^−1^) together with DFT-supported assignments, thereby extending both the spectral coverage and the interpretive detail relative to prior work. Where the spectral windows overlap, our 785 nm features are consistent with the bands observed by Malyshev et al., and we supply the corresponding assignments that were previously unavailable.

### 3.1. Raman Spectrum Analysis

**Figure 2:**
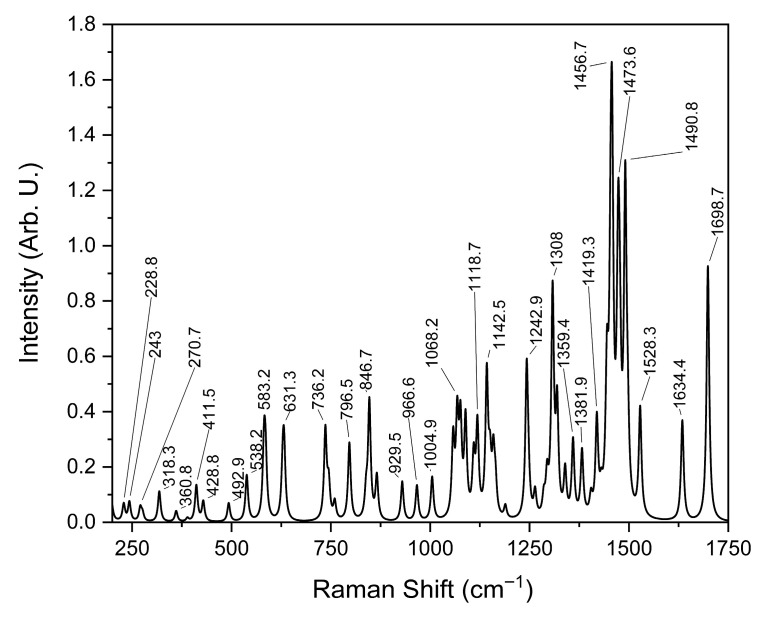
Simulated ADMA Raman Spectrum.

The simulated Raman spectrum of ADMA presents well-defined and detailed vibrational bands, providing a high-resolution theoretical reference for experimental comparison. As shown in Figure 2, the computational model predicts numerous characteristic peaks, which can be assigned to specific molecular vibrations.

**Figure 3:**
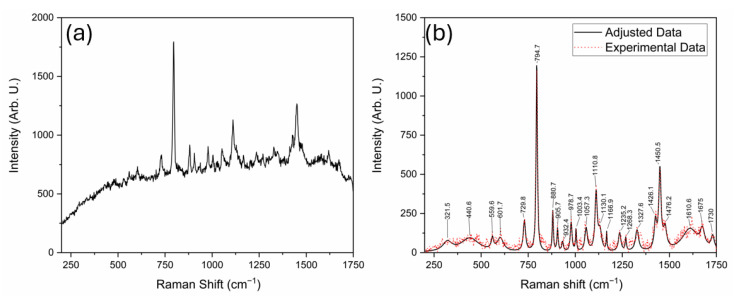
Raman spectrum of ADMA obtained using the 532 nm laser. Subplot (**a**) raw spectrum and subplot (**b**) background-free and adjusted spectra.

**Figure 4:**
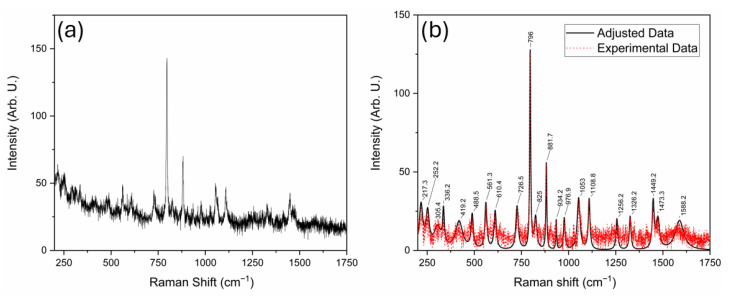
Raman spectrum of ADMA obtained using the 785 nm laser. Subplot (**a**) raw spectrum and subplot (**b**) background-free and adjusted spectra.

In contrast, the experimental Raman spectra obtained using 532 nm and 785 nm laser lines (Figure 3 and Figure 4, respectively) display broader features with varying degrees of intensity and noise. In these figures, subplots (a) stand for the raw spectra and subplots (b) stand for the background-free spectra. Several bands observed in the simulated spectrum are also present in the experimental data; however, notable differences in peak intensities and signal-to-noise ratio are evident. The spectrum acquired with the 785 nm laser exhibits a higher noise level, since from this signal we obtained an SNR of 95. In contrast, on 532 nm, we obtained a 136.5 SNR, which may obscure weaker vibrational signals and reduce spectral resolution. Nevertheless, this wavelength minimizes fluorescence background, making it advantageous for detecting broad vibrational features. The comparison of 532 nm and 785 nm excitation demonstrates the expected λ^−4^ dependence of Raman scattering intensity. Although the longer wavelength minimizes fluorescence, it also decreases the scattering cross-section, resulting in weaker but cleaner signals. This observation confirms the correct physical scaling behavior of ADMA spectra and validates the experimental configuration for subsequent biosensing adaptation [25]. Conversely, the 532 nm laser produces a cleaner spectrum with sharper and more intense peaks, but is more susceptible to fluorescence interference from biological compounds, as shown in Figure 3a, where fluorescence is more prominent than for the 785 nm laser case shown in Figure 4b. The curve labeled “Adjusted Data” in both subplots (Figure 3b) and Figure 4b represents a mathematical treatment applied using the Fityk 1.3.1 software. This fitting process was implemented to reduce noise in the experimental measurements and assist in elucidating vibrational modes corresponding to the ADMA molecule. The analysis was carried out by first applying a baseline correction using the built-in function in Fityk software, followed by fitting the spectrum with functions corresponding to the height and width of each identified band.

Table 1 summarizes the feature metrics of observed Raman bands across the experimental and simulated spectra. Each band is assigned to a corresponding vibrational mode and associated chemical bonds, aiding in interpreting and validating the spectral data obtained using the 532 nm laser. As observed, notable similarity exists between the experimental data and the adjusted curve, with similarity values greater than 0.9. However, the comparison between the simulated spectrum and both the fitted and experimental curves yields values below 0.6, indicating moderate to low similarity. This may be because the DFT spectrum corresponds to the isolated molecule, whereas the measurements were acquired on solid ADMA. Solid-state effects like intermolecular hydrogen bonding and crystal packing, protonation/conformer distributions, and anharmonicity can shift bands and alter relative intensities. Also, preprocessing (baseline/normalization) may further reduce feature-metric agreement. Nevertheless, a high proportion of the bands predicted by DFT can still be recovered from the experimental spectra, as shown later in this section.

**Figure 5:**
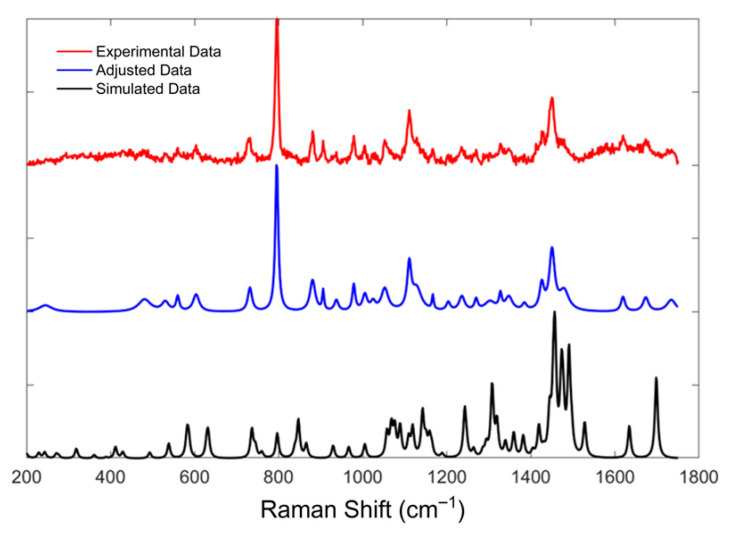
Normalized Raman spectra of ADMA obtained from experimental data, fitted, and simulated data.

Figure 5 presents the normalized Raman spectra for experimental data, simulation, and the adjusted curve to explain this behavior further. The vibrational modes in all three spectra occur approximately at the same Raman shift values. Nonetheless, the simulated spectrum exhibits significant differences in intensity, which likely account for the feature metrics below 0.6 reported in Table 1.

The comparison of Raman bands across the simulated and experimental spectra of ADMA, summarized in Table 2, provides detailed insights into the molecular vibrational behavior of this biologically relevant compound. Thirty vibrational bands were analyzed across three data sources: the simulated Raman spectrum, the spectrum acquired using a 785 nm laser, and the one obtained with a 532 nm laser. This comprehensive comparison allowed the tentative assignment of most bands to specific vibrational modes, including C–C and C–N stretching, N–H wagging, CH_2_ twisting, and asymmetric scissoring. In agreement with the trends reported by Sjöberg et al. [26] for tripeptide systems, ADMA’s experimental bands exhibited slight shifts compared to theoretical predictions, generally within 10–20 cm^−1^. This is particularly evident in bands located near 726, 880, and 1450 cm^−1^, which appear consistently across all spectral sources and are associated with highly localized and structurally preserved vibrations such as C=O and N–H modes. Interestingly, several simulated bands were not clearly observed in the experimental spectra, particularly in the higher wavenumber range (e.g., 1381.9 and 1698.7 cm^−1^). These discrepancies may result from fluorescence-induced signal attenuation or intrinsic differences in Raman activity under varying laser excitation conditions [27].

As observed in prior studies of amino acid-based systems, such deviations in intensity are common when comparing theoretical predictions to experimental results due to environmental effects and limitations in modeling absolute Raman intensities. Moreover, the presence of strong and well-defined bands between 600 and 1300 cm^−1^ confirms the existence of characteristic skeletal vibrations and side-chain motions. Bands such as 929.5, 1004.9, and 1242.9 cm^−1^ can be confidently attributed to coupled C–C and N–H deformations, consistent with previous assignments in vibrational studies of arginine analogs and peptide fragments [28]. A notable observation is the co-localization of band positions across the spectra, which suggests that despite variations in intensity, the molecular framework of ADMA is preserved and identifiable through Raman spectroscopy. This is particularly relevant for diagnostic applications, where structural fingerprints rather than intensity patterns may offer more robust detection criteria. The interpretation of vibrational modes was facilitated by DFT calculations, which provided not only a high-resolution theoretical spectrum but also allowed the visualization and decomposition of vibrational normal modes. These results underscore the importance of combining experimental techniques with theoretical simulations for the reliable assignment of vibrational features in complex biomolecules. Overall, the consistency in band positions, supported by theoretical assignments and spectral alignment, confirms the applicability of Raman spectroscopy for ADMA identification. The data presented here contribute to establishing a spectroscopic baseline for detecting ADMA in biological or clinical samples, supporting its role as a diagnostic biomarker. These results demonstrate the complementarity of theoretical and experimental approaches for the vibrational characterization of ADMA and highlight the relevance of combining Raman spectroscopy with density functional theory (DFT) simulations for molecular identification.

**Table 2:** Assignment of vibrational Raman bands of ADMA based on experimental and simulated spectra.

Simulated Bands	Bands Using a 785 nm Laser	Bands Using a 532 nm Laser	Assignments
228.8	217.2		ω C-H; ρ N-H
243	252.2		τ N-H
270.7			γ Molecular
318.3	305.4	321.5	γ Molecular
360.8	336.2		γ Molecular
411.5	419.2		γ Molecular
428.8		440.6	γ Molecular
492.8	488.5		ρ Molecular
538.2		559.6	ρ NH2; τ C-O, C=O
583.2	561.3		δ N-C; γ C-H; ω N-H; ω C-O
631.3	610.4	601.7	δ C-O; ρ C-H; γ Molecular
736.2	726.5	729.8	ν C-C; δ C-O; τ C-H
796.5	796.1	794.7	ω N-H, C-N; τ C-H
846.7	881.7	880.7	γ Molecular
929.5	934.2	932.4	ν C-C; ρ C-H; ω N-H
966.6	976.9	978.7	τ C-H; ν C-C; ν N-C; ν N-H, ν C-O
1004.9		1003.4	ν C-C; ρ N-H, C-H; ν C=N
1068.2	1053.1	1057.3	τ N-H; ν C-N, C=N: ρ C-H
1118.7	1108.1	1110.8	γ Molecular
1142.5		1166.9	γ Molecular
1242.9	1256.2	1235.2	τ N-H; τ C-H
1308			ω C-H; τ N-H, ν O-H, C-O
1359.4	1326.2	1327.6	γ Molecular
1381.9			γ Molecular
1419.3		1426.1	γ Molecular
1456.7	1449.2	1450.5	δ C-H; τ N-H
1473.6		1476.2	δ C-H
1528.3			ρ C-H; τ C-H; δ C-H; ν C-H
1634.4		1610.6	δ N-H; δ O-H; δ C-H
1698.7		1675	ν C=NH; δ C-N-H; ν N-C; δ C-H

ν, stretching; δ scissoring; ρ, rocking; ω, wagging; τ twisting; γ general bending [15,16,29,30].

### 3.2. FT-IR Spectrum Analysis

**Figure 6:**
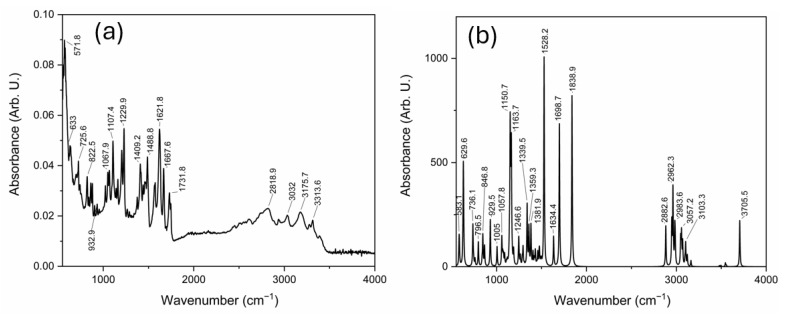
FT-IR spectrum of ADMA: (**a**) experimental absorbance, and (**b**) simulated absorbance.

The experimental absorbance FT-IR data were acquired using the ATR accessory, which facilitates the collection of spectra from a wide range of materials, including ADMA in its lyophilized form. The experimental spectrum is presented in Figure 6a, while the simulated spectrum obtained through DFT calculations is shown in Figure 6b. The simulated spectrum exhibits sharper and more defined peaks, reflecting the idealized conditions and higher resolution of the theoretical model. In contrast, the experimental spectrum displays broader bands and lower intensity in certain regions, which can be attributed to instrumental limitations and sample-specific factors such as physical state and intermolecular interactions.

The comparison between simulated and experimental FT-IR data is detailed in Table 3, where the main vibrational bands are listed along with their corresponding assignments.

Notably, despite the differences in resolution and intensity, key vibrational modes—such as the N-H scissoring around 1621 cm^−1^ and the C=NH stretching near 1667 cm^−1^—were successfully identified in both spectra. It is essential to highlight that the simulations represent an ideal gas-phase molecule without external interactions. At the same time, the experimental measurements involve the solid-state form of ADMA, which introduces factors such as hydrogen bonding and intermolecular forces that can shift or broaden the observed bands [31]. Additionally, the literature reports on ADMA embedded in organic matrices suggest further spectral shifts and intensity variations due to complex interactions with the host material. For instance, Malyshev et al. [25] demonstrated that incorporating ADMA into organic semiconductor-based molecularly imprinted polymer films induces noticeable changes in the vibrational response, particularly in Raman spectra, attributed to matrix-induced electronic and steric effects. In our case, the isolated ADMA molecule allows for a more straightforward assignment of vibrational modes without interference from external interactions, providing valuable reference data for future studies involving ADMA in more complex environments. The FT-IR results demonstrate similarity between the theoretical predictions and experimental data, particularly in the functional group region. This comparison validates the computational model and supports its use for accurate vibrational mode assignments. Beyond the comparative analysis, these results also provide a practical framework for future sensing applications. The accurate assignment of characteristic vibrational bands, particularly the N–H scissoring near 1621 cm^−1^ and the C=NH stretching around 1667 cm^−1^, defines reliable spectral markers that can serve as calibration references in optical detection schemes. Such bands, being both intense and structurally specific, are promising for use in Raman- or FT-IR-based biosensors aimed at ADMA quantification in complex media. In this sense, the present work establishes the spectroscopic groundwork required to design and calibrate label-free detection platforms, bridging fundamental vibrational characterization with its eventual analytical implementation.

## 4. Applied Perspective and Future Work

The Raman/FT-IR assignments presented here establish clear spectral regions of interest that can be translated into optical sensing workflows for ADMA. Our immediate goal is to validate these features in progressively more complex biological matrices, using consistent preprocessing and standard calibration to obtain practical figures of merit like sensitivity, robustness, and transferability. Where native Raman signals are weak, surface-enhanced Raman spectroscopy (SERS) will be employed to amplify band intensities at the assigned regions, preserving the same feature targets while improving detectability. Only targeted theoretical refinements will be applied where matrix effects cause systematic shifts, converting the present reference assignments into deployable measurements for real-world detection. Recent advances in fiber-integrated SERS and in microfluidic ATR-FT-IR with chemometric calibration demonstrate label-free, quantitative biosensing in liquid matrices, supporting the direct translation of our assigned regions into deployable ADMA assays [32,33,34].

## 5. Conclusions

N^G^, N^G^-dimethylarginine (ADMA), an endogenous compound closely linked to metabolic, cardiovascular, and renal diseases, was successfully characterized through a vibrational spectroscopy methodology. Both Raman and FT-IR spectra were experimentally acquired, and a comprehensive computational model was developed using Density Functional Theory (DFT) with the B3LYP functional and the cc-pVDZ basis set, implemented in the GaussView (version 5.0.8) and Gaussian 09 software packages. The comparison between experimental and simulated spectra, illustrated in Figure 2, Figure 3, Figure 4, Figure 5 and Figure 6 and summarized in Table 1, Table 2 and Table 3, provided more profound insights into the vibrational behavior of ADMA and enabled detailed band assignments. Notably, up to 86.7% of the Raman bands and 54% of the FT-IR bands were successfully identified and assigned to specific vibrational modes, demonstrating the robustness of the combined experimental-computational approach. The Raman spectra revealed good positional agreement and concordance of characteristic bands across experimental and simulated data, despite intensity discrepancies attributable to instrumental limitations and environmental effects. FT-IR analysis further confirmed the presence of key functional group vibrations, with excellent correlation in the fingerprint region. The successful identification of prominent bands, such as the N–H scissoring near 1621 cm^−1^ and C=NH stretching at 1667 cm^−1^, emphasizes the reliability of the applied methodologies. Remarkably, the purified ADMA molecule studied here allowed for a precise assignment of vibrational modes without interference from matrix interactions, establishing a valuable spectral reference for future studies involving ADMA in more complex environments, including biological matrices. By delivering a complete set of Raman/FT-IR features with DFT-supported assignments, this study delivers a reference framework for ADMA that is directly actionable for biosensing development. Translation toward sensing contexts will require matrix-based validation and calibration, which we delineate in the Section 4.

## Figures and Tables

**Table 1:** Comparative analysis of pairwise similarity of ADMA Raman spectra.

Feature Metrics	Experimental vs. Adjusted	Adjusted vs. Simulated	Experimental Data vs. Simulated	
Cosine	0.9365	0.5688	0.5601	
Correlation	0.9021	0.3646	0.3078	

**Table 3:** Comparison of the simulated and experimental infrared spectra and the band assignments.

Simulated Bands	FT-IR Bands	Assignments
583.1	571.8	γ Molecular
629.6	633	ω O-H; ρ C-H; δ C-H; ν N-H
736.1	725.6	ν C-C; ρ N-H; δ C-O; ω C-H
796.5		ω N-H; τ C-H
846.8	840	τ C-H; δ N=C-N, O=C-O; ν C-N, C-C; ω N-H
929.5	933	ν C-C; τ C-H; ω N-H, C-H
1005		γ Molecular; ω N-H
1057.8	1067.9	γ Molecular; ω N-H, C-H
1150.7	1107.4	γ Molecular
1163.7		γ Molecular; τ N-H
1246.6	1229.9	γ Molecular; ω N-H
1339.5		γ Molecular; τ N-H
1359.3		γ Molecular; ω N-H
1381.9		γ Molecular; ω N-H
1404.5	1409.2	ω C-H; τ N-H; ν C-H
1490.7	1488.8	δ C-H; ν C-N
1528.2		ρ C-H, N-H; δ N-C
1634.4	1621.8	δ N-H; ρ C-H
1698.7	1667.6	ν C-N, C=N; δ C-H; ρ C-H
1838.9		ν C=N; ρ N-H, C-H; δ O-H, O-C
2882.6		ω C-H
2983.6		ν C-H
3057.2	3032	ν C-H
3103.3		ω C-H
3162.5	3175.7	ν C-H
3705.5		ν O-H

ν, stretching; δ, scissoring; ρ, rocking; ω, wagging; τ, twisting; γ general bending [15,16,28,29].

## Data Availability

Data are contained within the article.
